# Clinical presentation of COVID-19 – a model derived by a machine learning algorithm

**DOI:** 10.1515/jib-2020-0050

**Published:** 2021-03-04

**Authors:** Malik Yousef, Louise C. Showe, Izhar Ben Shlomo

**Affiliations:** Head of the Galilee Digital Health Research Center, Safed, 13206, Israel; Zefat Academic College, Safed, 13206, Israel; The Wistar Institute, Molecular and Cellular Oncogenesis Program, Philadelphia, PA, USA; Head of the Program of Emergency Medicine, Safed, Israel

**Keywords:** clinical presentation, COVID_19, machine learning, national registry, risk allocation

## Abstract

COVID-19 pandemic has flooded all triage stations, making it difficult to carefully select those most likely infected. Data on total patients tested, infected, and hospitalized is fragmentary making it difficult to easily select those most likely to be infected. The Israeli Ministry of Health made public its registry of immediate clinical data and the respective status of infected/not infected for all viral DNA tests performed up to Apr. 18th, 2020 including almost 120,000 tests. We used a machine-learning algorithm to find out which immediate clinical elements mattered the most in identifying the true status of the tested persons including age or gender matter, to enable future better allocation of surveillance policy for those belonging to high-risk groups. In addition to the analyses applied on the first batch of the available data (Apr. 11th), we further tested the algorithm on the independent second batch (Apr. 12th to 18th). Fever, cough and headache were the most diagnostic, differing in degree of importance in different subgroups. Higher percentage of men were found positive (9.3 vs. 7.3%), but gender did not matter for the clinical presentation. The prediction power of the model was high, with accuracy of 0.84 and area under the curve 0.92. We provide a hand-held short checklist with verbal description of importance for the leading symptoms, which should expedite the triage and enable proper selection of people for further follow-up.

## 1 Introduction

COVID-19 pandemic has flooded triage stations with people seeking to verify whether they are infected. In this situation, the proper selection of those most likely infected is crucial, since one wishes to mark those who belong to high-risk groups, in order to rapidly offer them a closer surveillance, even before laboratory test results become available. A recent meta-analysis cited 3 models that addressed this goal. All models used less than 2,000 tests on suspected or asymptomatic people [1]. An important point in the coverage of data on patients rather than total tested vs. positive people is the fact, that reporting institutes are mostly hospitals [2]. We did not find a formal governmental publication reporting total tested vs. infected with any clinical data. When numbers exist on total testing vs. infected, they are not tabulated in relation to symptoms. For example, in Great Britain until Apr. 17, 357,023 people were tested, among whom 114,217 (32%)) were positive, a percentage representing most probably a stringent policy of testing, allowing relatively highly suspected people to be tested.

The Israeli Ministry of Health made public its registry of immediate clinical data and the respective status of infected/not-infected for 107,542 laboratory-tested people recorded until April 11, 2020. Among these, 8,956 (8.3%) tested positive and 98,586 negative.

We used a machine-learning algorithm to determine which clinical elements were most critical to determining the true COVID-19 status, including age or gender in order to enable a policy that would provide for a rapid identification of individuals belonging to groups at high-risk of infection. We further tested the algorithm developed on new independent data reported from Apr. 12th to 18th.

## 2 Data

At the early days of the pandemic the Israeli government has created a web database for COVID-19 that is posted in the Data.gov.il official website [3].

The database consists of different datasets. For our current study we have considered the dataset for the people tested for COVID_19 viral DNA. The data consist of 10 tabulated features for each participant. All the values of this data are categorical.

The data we have downloaded related to the last update on April 12 2020 (records for tests until April 11). The data consist of 126,067 tested persons. We have removed all person’s rows that include at least one NULL (missing value) to yield 124,466 tests, which contains 114,226 negative test and 10,240 positive tests. Negative test means no viral DNA detected. We will refer to this data as batch one. Additional cleaning of the data was performed to remove all zero rows belonging to the “pos” class. We consider just the five features (fever, cough, headache, sore throat and shortness of breath) to determine zero rows. Zero row is a row that consist of just zero values. Our assumption that this kind of test is wrongly assigned to be infected samples, or it might be that the reason of infection is not determine by the current set of features used in the data. After this step, we have 10,513 tests with 6,427 being positive and 98,586 negative.

During our research, a new batch of the data was posted that reports data from April 12, 2020 until April 18, 2020. We refer to it as batch two data. This batch also was subject to the step of removing zero rows belonging to class pos. The new batch consist of 16,156 tests where 499 are positive and 15,657 are negative. This includes, molecular databases, information systems and data warehouses, integration of data (methods and tools), metabolic and regulatory network modeling and simulation, signal pathways and cell control, network analysis, medical informatics, biomedicine and biotechnology, integrative approaches for drug design as well as integrative data and text mining approaches.

### 2.1 Training and evaluation of the model

We used the random forest (RF) [4] classifier implemented by the platform KNIME [5]. The RF also provides a score that expresses the significance of each feature. Features in this study are the elements listed in Table 1. Significant features are important in building the final model. Features with a very low score could be discarded from the final model. This process is called feature selection in machine learning lingo. One aim of feature selection is the simplification of models in order to make them easier for interpretation.

**Table 1:** Immediate clinical features recorded for people sampled for viral DNA in Israel.

Parameter	Value	Details
Test date	dd/mm/yy	Day received in the lab
Gender	M/F/unknown	
Corona result	P/N/unknown (+at work)	The outcome of the COVID-19 test
Age ≥ 60	Y/N/unknown	Value is 1 if ≥60; 0 otherwise
Cough	Y/N/unknown	Before the test
Fever	Y/N/unknown	Before the test
Sore throat	Y/N/unknown	Before the test
Shortness of breath	Y/N/unknown	Before the test
Headache	Y/N/unknown	Before the test
Test indication	Abroad/Contact with infected/Other	Arriving from abroad? Contact with infected person? Other

**Table 2:** National Israeli COVID-19 DNA tests performed until Apr. 11, 2020, without the “Test Indication” feature (first batch). Average results obtained from 100 MCVV.

Ratio	#pos	#neg	Acc	Sen	Spe	Prec	F1	AUC
01:1	6,427	6,428	0.929	1.000	0.859	0.876	0.934	0.959
01:2	6,427	12,855	0.906	0.998	0.860	0.781	0.876	0.963
01:3	6,427	19,282	0.902	0.950	0.886	0.740	0.829	0.966
Stdv			0.007	0.011	0.013	0.015	0.008	0.004
Random labels (ratio 1:2)			0.36	0.98	0.05	0.34	0.51	0.51
Tests on second batch data (Apr. 12th to 18th 2020)			0.84	0.82	0.84			0.92

Ratio column describes the ratio between the positives and the negatives. Acc – accuracy, Sen – sensitivity, Spe – specificity, Prec – precession, F1 – F1-measure and AUC – area under the curve. “stdv” is the average of standard deviations for each corresponding measurement. The last row is the result for the same data while shuffling the labels of the data (random labels). Last row refers to the results obtained from deploying the model from the first batch on the second batch of the data (data recorded Apr. 12th to 18th).

**Figure 1:**
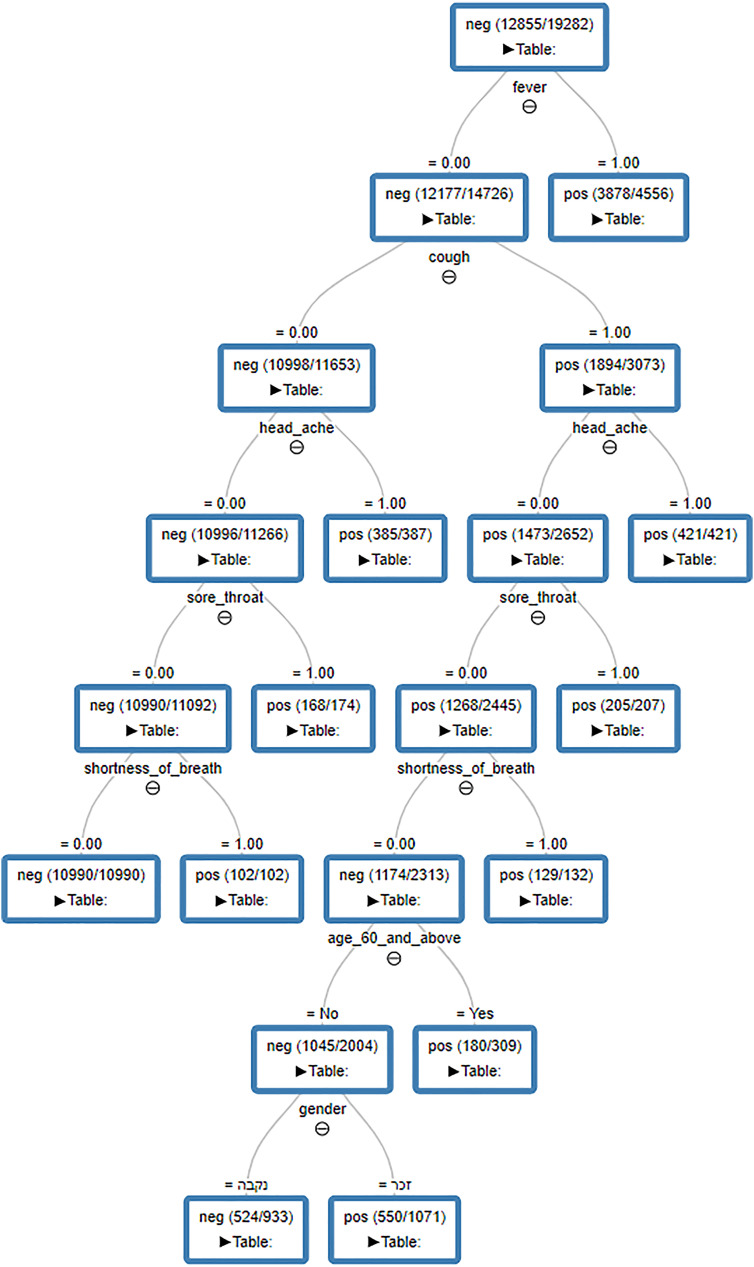
The decision tree by immediate clinical data obtained from applying the DT on all the first batch data published by the Ministry of Health on detection of viral DNA of COVID-19 up to Apr. 11, 2020, applied on the dataNonTI of ratio 1:2.

The RF classifier is an ensemble of decision trees. For visual simplicity, we present only the decision tree model applied on the whole data excluding the feature “Test indication” (Results including this feature are in the Supplemental Data).

For each experiment we have generated the decision tree (DT) based on the data. The DT model can be expressed as a set of if-then-else decision rules. The DT is a tree with decision nodes and leaf nodes. A decision node has two or more branches. A leaf node represents a classification or decision (“pos” or “neg” label). The topmost decision node in a tree corresponds to the best predictor called root node. For interpretation of DT one needs to start from the root node, moving to the next node based on the edges expression. This process is repeated until reaching the leaf node, the leaf node tells the prediction outcome (“pos” or “neg”). One can consider the path from the root to the leaf as rules connected by an “and” relationship.

The classifier was trained and tested with a split into 90% training and 10% testing data. While performing a 100-fold Monte Carlo cross-validation (MCCV) [6] for model establishment.

The features for each RF model were recoded over all the 100 iterations. The average of those scores were calculated to assign final score to each feature. The higher the score the more significant is the feature to the model.

In order to allow evaluation of the performance of the RF classifier, a set of the following measures are considered: (1) sensitivity (SEN) which represents the true positive rate, (2) specificity (SPE) which represents the true negative rate (complement of sensitivity), (3) precision (PREC) which represents the ability to correctly predict positive target condition to the total, (4) accuracy (ACC) represents the classifier ability to predict the target condition correctly, (5) F-measure represents the classifier ability to predict the target condition correctly (comparing to ACC, it tells a lot more in case of imbalanced data set, since it considers both PR and SE). The area under the ROC curve measurements (AUC) [7] is an estimate of the probability that a classifier will rank a randomly chosen positive instance higher than a randomly chosen negative instance.

All reported performance measures refer to the average of 100-fold Monte Carlo cross validation (MCCV) [6].

Sensitivity = TP/(TP + FN) (SEN, recall)
specificity = TN/(TN + FP) (SPE)
precesion = TP/(TP + FP) (PREC)
accuracy = (TP + TN)/(TP + FN + TN + FP) (ACC)
F-measure = 2 × (PR × SE)/(PR + SE) (F1)

After applying the process on data collected until Apr. 12, 2020, we tested it on the new data accumulated from 13th to 18th of April.

## 3 Results

We have generated and tested two models form of the given data (first batch). In one we considered all the features, whereas in the second one we excluded the “Test indication” feature that represents information about the source of the infection in case it happened, but not about the examinee. However, due to the new situation wherein the government almost cracked down all the international incoming flights, we wanted to suggest a model that is not affected by this feature. For simplicity we will refer to the data without the “Test Indication” feature by dataNonTI while the one with the Test indication with dataTI (details in Supplemental Data). Table 1 details the clinical parameter recorded.

Table 2 present the average performance results applied on dataNonTI. As the data is unbalanced, we have considered different ratio between the small class of positives and the large class of negatives. Ratio of 1:2 means that we took twice the number of random samples from the negative pool for each iteration from the 100 MCCV. Figure 1 present the decision tree obtained from applying DT on the dataNonTI of ratio 1:2. This tree of decision tells several things, which can help shape the right medical response.

1. Those with fever >38 °C, 85% were infected.
2. No fever but headache, practically all infected.
3. No fever, no cough, no headache but sore throat over 955 infected.
4. No fever, no cough, no headache, no sore throat but shortness of breath, all infected.
5. Fever, cough, and headache, all infected.
6. Fever, cough, no headache, but sore throat, all infected.
7. Fever, cough, no headache, no sore throat, but shortness of breath, all infected.
8. Fever, cough, no headache, no sore throat, no shortness of breath but 60 or older, 60% infected.

The total people arriving from abroad were 9,887, of which 1,356 (13.7%) tested positive for viral DNA. The respective numbers for home people were, 97,655 and 8,956 (9.2%). Since the mixes were profoundly shaped by almost no fever cases from abroad, the tree does not split the same for all nodes (Supplemental Data).

In general, as illustrated in Table 2 the low standard deviations indicate that the suggested model has a very high confidence. Table 3 present the ranks/score of significant of each feature.

**Table 3:** The ranks/score of the significance of each clinical feature on the National Israeli COVID-19 DNA tests assigned by RF model on the first batch of the data (until Apr. 11, 2020).

Feature	Score
Fever	1
Cough	0.643832075
Headache	0.462187066
Sore throat	0.227116751
Shortness of breath	0.110647828
Gender	0.009394193
Age ≥ 60	0.007142431

After the conclusion of the above analyses on first batch of the data, we tested the algorithm on the new data (second batch). A total of 16,156 new people were tested, among whom 499 (3.1%) were positive, a lower percentage than before. Yet, our testing indicated performance indices not worse than the constituting round (last row on Table 2). This result is a validation to our model that was created based on the first batch of the data. Table 4 provides a practical checklist for those manning the triage station, which should expedite decisions on where to send the patient.

**Table 4:** A check list for triage of patients seen for possible COVID_19 infection.

Feature	Likelihood of infection
1. fever	Very high
2. cough	Both present – absolute
3. headache	
4. sore throat	Absolute
5. shortness of breath	Absolute
6. gender	Marginal
7. age ≥ 60	More likely to be

## 4 Discussion

Physicians at junction points, specifically during stressful days, need clear protocols and guides to navigate their way with patients. In a recent meta-analysis Wynants et al. (2020) admitted that all 31 prediction models they reviewed were found to have a high risk of bias, and evidence from independent external validation of these models is currently lacking. More specifically, the models that evaluated prediction of infection as related to clinical data all dealt with less than a few thousands of cases. Our sample’s advantage is its being national record with about 120,000 samples and related immediate clinical elements. Even though one cannot dismiss the possibility of bias, the larger the sample and the larger population it covers, the less likely it is to be biased. Moreover, our test round over the 16,156 new tests, with only 3.1% positives indicates that our DT is robust and holds true for varying mixes of positives and negatives. We believe that our results make clear the correct way for the current COVID-19 days.

It is obvious that the data is not clean in the sense, that sampled populations were selected and not random. On the other hand, the high levels of personal stress, associated with crisis days, brings many unjustified cases to be tested, and a clear algorithm, even if somewhat rough, can help greatly.

The next step to this observation should be to align it against background data on the participants from the computerized records at the community clinics as well as hospitals. Such an amalgamation can provide even sharper tools not only to select the right immediate clinical decision but also provide an outlook for future patients.
