# Supplementary material for: Clinical presentation of COVID-19 – a model derived by a machine learning algorithm
Source: J Integr Bioinform. 2021 Mar 4;18(1):3–8. doi: 10.1515/jib-2020-0050 (PMC8035960; doi:10.1515/jib-2020-0050)
Supplement: Supplementary file 1 [file jib-18-20200050-s001.docx]

**Supplemental Data**

The total people arriving from abroad were 9887, of which 1,356 (13.7%) tested positive for viral DNA. The respective numbers for home people were, 97,655 and 8956 (9.2%). Since the mixes were profoundly shaped by almost no fever cases from abroad, the tree does not split the same for all nodes.

Introducing the feature “Test indication” to the analysis, effected the results dramatically as illustrated in Supplemental Table 2. The performance of the RF is increased by about 6% considering the balancing ratio of 1:2.

**Supplemental Table 1**. National Israeli COVID-19 DNA tests performed until Apr. 11, 2020 including the "Test Indication" feature. Average results obtained from 100 MCVV. Ratio column describes the ratio between the positives and the negatives. Acc - Accuracy, Sen - sensitivity, Spe - specificity, Prec - precession, F1 - F1-Meausre and AUC - area under the curve. "stdv" row is the average of standard deviation for each corresponding measurement. The last row is the result for the same data while shuffling the labels of the data (random labels).

| **ratio** | **#pos** | **#neg** | **Acc** | **Sen** | **Spe** | **Prec** | **F1** | **AUC** |
| --- | --- | --- | --- | --- | --- | --- | --- | --- |
| 1:1 | 8956 | 8956 | 0.85 | 0.86 | 0.85 | 0.85 | 0.85 | 0.91 |
| 1:2 | 8956 | 17193 | 0.88 | 0.79 | 0.92 | 0.84 | 0.81 | 0.91 |
| 1:3 | 8956 | 26868 | 0.90 | 0.75 | 0.95 | 0.83 | 0.79 | 0.91 |
| stdv | | | 0.01 | 0.02 | 0.01 | 0.02 | 0.01 | 0.01 |
| random labels (ratio1:02) | | | 0.34 | 0.99 | 0.02 | 0.34 | 0.50 | 0.50 |

**Supplemental Table 2**. Order of importance for immediate clinical features in prediction infection with COVID-19 as calculated from the National Israeli COVID-19 DNA tests performed until Apr. 11, 2020 (first bacth), as reported by the Ministry of Health.

| **feature** | **Significance ranking for dataNonIT** | **Significance ranking for dataIT** |
| --- | --- | --- |
| test_indication | nan | 1 |
| fever | 1 | 2 |
| headache | 3 | 3 |
| cough | 2 | 4 |
| Sore throat | 4 | 5 |
| Shortness of breath | 5 | 6 |
| Gender | 6 | 7 |
| age_60_and_above | 7 | 8 |

The DT model present in the Supplemental Figure is more complicated than the DT model in Figure 1 of the main text.


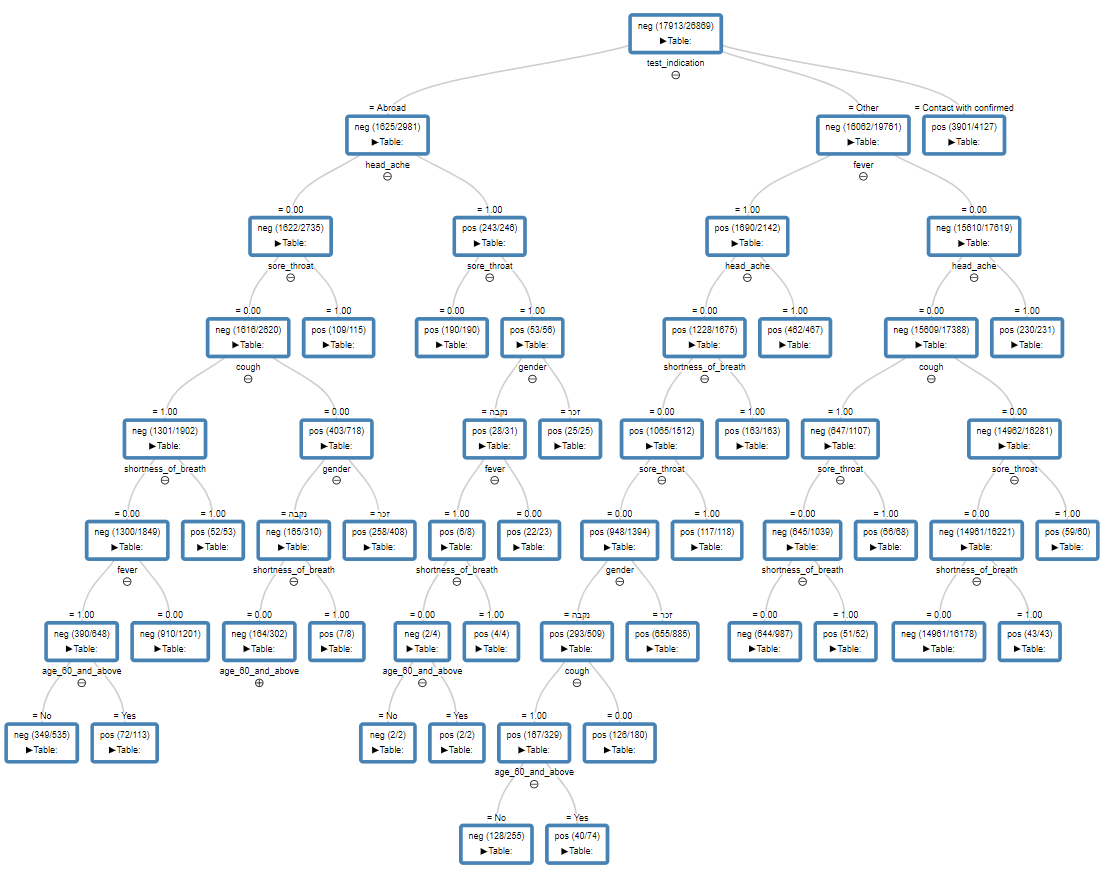


**Supplemental Figure**. The decision tree by immediate clinical data obtained from analyzing all the data published by the Ministry of Health on detection of viral DNA of COVID-19 up to Apr. 11, 2020 (first batch), applied on the dataTI of ratio 1:2.

It is obvious from the DT that coming from abroad takes prime role and that the feature of fever is ranked lower that among locals, as a reflection of the fact that people with fever were already not allowed to board the aircrafts.
